# A comparison of opioid overdose risk behaviors by race and ethnicity among overdose survivors in Boston, MA, and San Francisco, CA

**DOI:** 10.1186/s12954-026-01451-9

**Published:** 2026-04-20

**Authors:** Gabriela E. Reed, Vanessa M. McMahan, Yi-Shin Grace Chang, Natrina L. Johnson, Alexander Y. Walley, Phillip O. Coffin, Miriam T. H. Harris

**Affiliations:** 1 Division of General Medicine and Clinical Epidemiology, Department of Medicine, University of North Carolina School of Medicine, Chapel Hill, NC USA; 2 Grayken Center for Addiction and Clinical Addiction Research and Education (CARE) Unit, Section of General Internal Medicine, Boston Medical Center and Boston University School of Medicine, Boston, MA USA; 3 Center On Substance Use and Health, San Francisco Department of Public Health, San Francisco, CA USA; 4 Department of Population Medicine, Harvard Medical School and Harvard Pilgrim Health Care Institute, Boston, MA USA; 5 Department of Medicine, Division of Health and Society, Benioff Homelessness and Housing Initiative, University of California, San Francisco, San Francisco, CA USA; 6 Department of Medicine, University of California, San Francisco, San Francisco, CA USA

**Keywords:** Overdose, Race, Ethnicity, Risk behaviors, Drug use

## Abstract

**Background:**

Black and Latino people are at high risk for overdose mortality. Interventions focused on overdose risk behaviors may offer opportunities to reduce overdose, but little research has addressed racial and ethnic differences in risk behaviors. We explored differences in risk behaviors among Black, Latino, and White individuals who used opioids and had a history of prior overdose.

**Methods:**

We used cross-sectional baseline data from REBOOT 2.0 (the REpeated dose Behavioral intervention to reduce Opioid Overdose Trial) based in Boston, MA, and San Francisco, CA, which enrolled people with opioid use disorder and a history of opioid overdose. We used the Andersen behavioral model to inform covariate selection for a multivariable logistic regression analysis examining associations between race/ethnicity (Black, Latino, and White) and three individual overdose risk behaviors (using opioids alone, not using a tester dose, and using alcohol and/or benzodiazepines on the same day as opioids).

**Results:**

Of 247 participants, 19% were Black, 16% were Latino and 66% were White. More than 20% in each group reported each risk behavior. In unadjusted analyses, Black participants had higher odds of using alone (OR = 2.17, 95% CI 1.08–4.36) and lower odds of not using a tester dose (OR = 0.48, 95% CI 0.24–0.93), indicating greater tester dose use. After adjusting for age, gender and structural determinants of health (e.g., housing, incarceration, education, opioid use disorder treatment) the adjusted odds of Black participants using alone (aOR = 2.05, 95% CI 0.99–4.26) and a tester dose (aOR = 0.56, 95% CI 0.28–1.13) were no longer statistically significant.

**Conclusions:**

Overdose risk behaviors were common among Black, Latino, and White overdose survivors in San Francisco and Boston. Overdose risk behaviors were not significantly different for Black or Latino compared to White overdose survivors after adjusting for age, gender, and structural determinants of health. For individuals at the highest risk of overdose, individual prevention efforts remain necessary but are not sufficient. Interventions should also address the social-structural factors shaping overdose risk, including housing, criminalization, and access to treatment.

**Trial registration:**

Details found at clinicaltrials.gov, NCT02093559.

**Supplementary Information:**

The online version contains supplementary material available at 10.1186/s12954-026-01451-9.

## Background

While the overall age-adjusted rate of drug overdose deaths in the United States decreased between 2022 and 2023, this decline was not experienced equally across racial and ethnic groups. Among White people, the rate decreased from 35.6 to 33.1 deaths per 100,000, while the rate increased among Black people from 47.5 to 48.9 deaths per 100,000, remained stable among Latino people[1]^1^ (22.7 to 22.8 deaths per 100,000), and increased among American Indian and Alaskan Native people from 56.4 to 56.7 deaths per 100,000 [2]. Risk behavior interventions tailored to the unique needs of racialized communities may offer opportunities to reduce overdose risk of populations at high risk. Risk behaviors that increase the risk of overdose include using multiple sedating substances, such as opioids with alcohol and/or benzodiazepines and using opioids alone, reducing the chance of a bystander being able to respond and intervene in an overdose event. Conversely, protective behaviors include using a tester dose or small amount to “test” the drug being used [3–5].

However, there is a paucity of evidence and inconsistent results from prior studies examining racial and ethnic differences in overdose risk behaviors. A study of 196 participants across three US cities found that when fentanyl was suspected in their drug supply, Black and Latino participants were twice as likely as White participants to engage in protective overdose behaviors, such as using smaller amounts, taking turns using drugs, or obtaining naloxone [6]. However, this study grouped all non-White participants together rather than examining racial and ethnic participants separately, and analyzed protective behaviors as a composite outcome rather than individual risk behaviors [6]. Another study, examining 20 years of death records, showed that Black individuals had higher rates of combined alcohol and opioid-related deaths, while Latino individuals had lower rates of these deaths, compared to White individuals [7]. Among 509 individuals from Rhode Island, regular use of benzodiazepines was significantly higher among White participants compared to other racial and ethnic groups [8]. This study did not find other variations in drug use behaviors or harm reduction practices by race or ethnicity [8]. Given the inconclusive and limited nature of the existing literature, a more nuanced understanding of individual overdose risk behaviors is needed to address overdose inequities among racial and ethnic populations and inform tailored overdose prevention interventions.

To address this gap, this study explored differences in overdose risk behaviors among Black, Latino, and White individuals with a history of opioid use and previous overdose. Understanding overdose risk behaviors among those with a history of overdose is particularly important because overdose survivors have demonstrated vulnerability to overdose, are likely to continue risky drug use, and face an elevated risk of subsequent fatal overdose [9–11]. Moreover, the period following an overdose may represent a critical window for intervention, as survivors may be more motivated to adopt risk-reduction behaviors.

## Methods

### Study design and population

Data were derived from the REBOOT (REpeated dose Behavioral intervention to reduce Opioid Overdose) 2.0 study. REBOOT was a two-site randomized-controlled trial designed to assess the effectiveness of a repeated counseling intervention to reduce opioid overdose. This analysis used cross-sectional baseline data from the REBOOT 2.0 study. The study was approved by the University of California, San Francisco Institutional Review Board (#17–24,203).

Details of the REBOOT study design have been described elsewhere [12]. Briefly, adults, aged 18 to 65 years old, from Boston, MA, and San Francisco, CA, were consented and enrolled between April 2019 and June 2022. All participants had moderate to severe opioid use disorder per the criteria of the Diagnostic and Statistical Manual of Mental Disorders, Fifth Edition, reported non-prescribed opioid use in the past 14 days, reported opioid overdose in the past three years, had previously received take-home naloxone, and had an opioid-positive urine drug screen at enrollment (or third-party confirmation of opioid use for remote visits during COVID-19 restrictions). Data were collected at baseline as well as every four months over the 16-month study duration, either in-person or via telephone, and entered into REDCap by research staff [13, 14]. The study questionnaire included demographic data, substance use, overdose risk behaviors, and substance use disorder treatment experiences. Participants were compensated on an escalating scale between $20 (e.g., screening visit) and $100 (final study visit) for study participation. In this study, we analyzed data from the REBOOT 2.0 enrollment visit and included trial participants who self-identified as Black, Latino, or White.

### Outcome variables

We selected three individual overdose risk behaviors informed by the literature that are known to increase risk of overdose occurrence or fatality as our primary outcome measures [3, 15–18]: 1) using alone, defined as using opioids without someone else at the same time, 2) not using a tester dose, defined as the absence of a tester dose (i.e., not engaging in the protective behavior of using a smaller amount first or using slowly to assess drug strength), and 3) using opioids the same day as using alcohol and/or benzodiazepines. These behaviors reflect choices made at the time of drug use, which are contextualized by structural factors, such as policing and access to treatment, that shape both individual overdose behaviors and the broader drug‑use environment. We did not explore naloxone use because the trial required previous naloxone receipt for enrollment, which could bias results.

We combined co-use of alcohol and benzodiazepines as one of our primary outcome measures because both increase the risk of respiratory depression and sedation when used with opioids. However, existing literature indicates differences in co-use rates among Black and Latino individuals for alcohol versus benzodiazepines [7, 8, 19]. Combining the co-use of alcohol and/or benzodiazepines with opioids may obscure these racial differences. Therefore, we also conducted a secondary analysis to examine alcohol and benzodiazepine use separately on the same day as opioid use.

All behaviors were assessed over the preceding four months. How often individuals engaged in a risk behavior was assessed using a Likert scale of “never,” “rarely,” “often,” “sometimes,” or “always” when using opioids. For this analysis, behaviors were dichotomized into not engaged defined as “never” or “rarely” versus engaged defined as “sometimes,” “often,” or “always.” Outcomes were presented as the responses associated with increased overdose risk. For using alone and using alcohol or benzodiazepines on the same day as using opioids, sometimes, often, or always, were considered to increase overdose risk. For using a tester dose, never or rarely were considered to increase risk.

### Predictor variables

The independent variables were defined by self‑reported race and ethnicity and included the mutually exclusive categories of Black or White race, and Latino ethnicity. We developed mutually exclusive racial and ethnic categories to minimize overlap between groups and facilitate clearer comparisons, improving the interpretability of any variability found in overdose risk behaviors. Options for racial identity included Native American/Alaska Native, Asian, Native Hawaiian or other Pacific Islander, Black or African American, White, More than one race, or Other. Black participants were defined as those who self-identified as Black or African American, including Multiracial participants reporting Black or African American race. Options for ethnic identity included Hispanic/Latinx or non-Hispanic/Latinx. Latino participants were defined as those who self-identified as Hispanic/Latinx, inclusive of any race. White participants were defined as those who self-identified as White and non-Hispanic/Latinx. We excluded individuals who did not identify as Black, White, or Hispanic/Latinx, including American Indian and Alaska Native participants, due to insufficient sample sizes to support meaningful statistical inference.

### Conceptual framework

To understand how individual health behaviors may be impacted by race or ethnicity, it is critical to consider their structural context, specifically racism and the related inequities in social determinants of health and healthcare access [8, 20, 21]. Thus, we applied the Andersen behavioral model, a health equity framework, to account for structural racism in our exploration of individual-level overdose risk behaviors [22–24]. This model posits that individual deleterious or protective health behaviors are predicated upon a sequence of predisposing, enabling, and need factors. Predisposing factors may include demographic characteristics such as age and gender [25–27]. Enabling factors influence health service engagement, and include structural determinants such as housing, education, incarceration, and access to treatment [10, 28–30]. By situating individual‑level behaviors within their structural context, this framework allows us to assess whether racial and ethnic differences in overdose risk behaviors persist after accounting for structural inequities. Lastly, need factors impact the necessity for a health behavior; for example, the current use of non-prescribed opioids influences the need for protective overdose behaviors. Here, we explored whether overdose risk behaviors varied between Black, Latino, and White participants, in the context of the below predisposing (age and gender) and enabling (unstable housing, education, incarceration, location, and opioid use disorder (OUD) treatment) factors among a group of overdose survivors, with a need factor for overdose risk reduction (see Table 2 for greater detail on how variables were measured and operationalized). The predisposing and enabling factors were chosen based on clinical expertise and the literature.

### Predisposing and enabling variables

Characteristics included age (years), gender (male or trans male vs female or trans female), housing (stable vs unstable), education (less vs more than receipt of high school diploma or equivalent), incarceration (jail or prison in the previous four months, yes vs no), and location defined as the research site of participation (Boston vs San Francisco). OUD treatment was defined as receipt of medications for OUD (MOUD, yes vs no) in the past four months (taking methadone from a clinic, prescribed oral or long-acting injectable buprenorphine, or prescribed oral or injectable extended-release naltrexone).

### Descriptive variables

To further describe the cohort, we included the following characteristics: baseline HIV status, health insurance (private, public or Veterans Affairs, vs uninsured), and employment status (having a full or part-time job vs unemployed), and substance use defined by the frequency (reported as number of days out of past 30; categorized into daily use, some use, vs no use) and primary route (injection, smoking, nasal, vs oral) of opioid use (heroin, non-prescribed fentanyl, and/or other non-prescribed opioids/painkillers) and stimulant use (cocaine or methamphetamine/amphetamines). We also reported use of fentanyl test strips (never or rarely vs sometimes, often, or always) and buying opioids from a dealer you do not regularly go to (never or rarely vs sometimes, often, or always). These overdose risk behaviors were not included in comparative analyses because they are less well established in the literature. Other substance use disorder treatment characteristics included receipt of residential treatment (being in a residential treatment program, inpatient detox program, or sober house) and outpatient recovery counseling (attending AA/NA meetings or receiving outpatient counseling for substance use) in the past four months.

### Statistical analyses

We used descriptive statistics to summarize data, with counts and percentages for categorical variables and measures of central tendency for continuous variables. Since continuous data were skewed, we used medians and interquartile ranges.

For each individual overdose risk behavior outcome, models were built using the following steps based on the Andersen behavioral model’s conceptual framework. First, bivariate logistic regression analyses were used to assess the association of race, ethnicity, predisposing, and enabling factors and each outcome of using alone, not using a tester dose, and using alcohol and/or benzodiazepines on the same day as opioids. Next, adjusted multivariable logistic regression analyses were performed to assess the independent association of race and ethnicity with the three overdose risk behaviors. Model 1 included variables measuring predisposing factors (the demographic covariates of age and gender). Model 2 added enabling factors (research site, MOUD treatment, housing, incarceration, and receipt of high school diploma or equivalent). Lastly, we secondarily examined using alcohol or benzodiazepines on the same day as opioids separately to assess differences by race/ethnicity with the use of each of these substances independently. The significance level for all analyses was set at 0.05 and all analyses were performed using Stata 17 statistical software (StataCorp, College Station, TX).

## Results

Of 268 REBOOT participants, 247 were included in this analysis (21 did not identify as Black, Latino, or White); 66% were White, 19% Black, and 16% Latino (see Table 1). The median age was 42 years (IQR: 34–50) and 62% identified as male or trans male. At baseline, 64% were experiencing unstable housing, 10% had been incarcerated over the past four months, and 85% were unemployed. All participants reported non-prescribed opioid use in the past 30 days, of whom 54% reported daily use and 68% reported primary use by injection. Most participants used stimulants in the past 30 days (93%), of whom 35% reported daily use. Most (70%) had received MOUD in the past four months, 53% had engaged in outpatient recovery counseling, and 23% had undergone residential substance use treatment. Most reported using alcohol or benzodiazepines the same day as opioids (57%), 52% did not use a tester dose, and 27% had used alone in the past four months.

**Table 1:** Baseline characteristics of participants with a history of opioid overdose from Boston and San Francisco, 2019 – 2022 (N = 247*)

	Total n (%)	White participants n (%)	Black participants n (%)	Laine participantst n (%)
Total	247 (100)	162 (66)	46 (19)	39 (16)
Age – [median, IQR]	42 (34–50)	42 (34–49)	47 (38–56)	39 (35–49)
*Gender*				
Male or trans male	153 (62)	100 (62)	26 (57)	27 (69)
Female or trans female	94 (38)	62 (38)	20 (43)	12 (31)
*Site*				
San Francisco	142 (57)	96 (59)	23 (50)	23 (59)
Boston	105 (43)	66 (41)	23 (50)	16 (41)
Treated with MOUD ^a,b^	172 (70)	118 (73)	30 (65)	24 (62)
Unstably housed ^a,c^	158 (64)	104 (64)	29 (63)	25 (64)
Incarcerated ^a,d^	25 (10)	15 (9)	4 (9)	6 (15)
Completed high school/GED or above	217 (88)	147 (91)	43 (93)	27 (69)
HIV Positive ^e^	18 (7)	10 (6)	6 (13)	2 (5)
Currently insured	245 (99)	160 (99)	46 (100)	39 (100)
Currently employed	38 (15)	23 (14)	8 (17)	7 (18)
*Past 30-day cocaine or methamphetamine use*				
Daily use	80 (32)	50 (31)	16 (35)	14 (36)
Some use	150 (61)	104 (64)	23 (50)	23 (59)
No use	17 (7)	8 (5)	7 (15)	2 (5)
*Past 30-day opioid use* ^f^				
Daily use	134 (54)	79 (49)	28 (61)	27 (69)
Some use	113 (46)	83 (51)	18 (39)	12 (31)
No use	0 (0)	0 (0)	0 (0)	0 (0)
*Primary route of opioid use* ^a,f^				
Inject	167 (68)	116 (72)	24 (52)	27 (69)
Smoke	43 (17)	31 (19)	6 (13)	6 (15)
Sniff	36 (15)	14 (9)	16 (35)	6 (15)
Oral	1 (0)	1 (1)	0 (0)	0 (0)
Used fentanyl test strips ^g^	32 (13)	19 (12)	9 (20)	4 (10)
Purchased opioids from a new dealer ^g^	103 (42)	71 (44)	15 (33)	17 (44)
Residential treatment ^a,h^	58 (23)	43 (27)	9 (20)	6 (15)
Engaged in outpatient recovery counseling ^a,i^	131 (53)	85 (52)	27 (59)	19 (49)
Used alcohol and/or benzodiazepines on the same day as opioids ^a,g,l^	142 (57)	95 (59)	26 (57)	21 (54)
Did not use a tester dose ^a,j^	129 (52)	93 (57)	18 (39)	18 (46)
Used alone ^a,k^	66 (27)	37 (23)	18 (39)	11 (28)
Used alcohol and opioids on the same day ^a,g^	72 (29)	93 (57)	19 (41)	9 (23)
Used benzodiazepines and opioids on the same day ^a,g^	104 (42)	37 (23)	10 (21)	16 (41)

^*^21 individuals from the original study cohort were excluded

^a^ Variable assessed over the past four months

^b^ MOUD defined as taking methadone from a methadone clinic for OUD, taking Suboxone prescribed by a doctor, receiving Sublocade injection by a doctor, receiving Vivitrol injection by a doctor, or taking naltrexone prescribed by a doctor

^c^ Unstable housing operationalized as spending any nights on the street or in a homeless shelter

^d^ Incarceration defined by self-report

^e^ Two individuals had unknown HIV status

^f^ Reported as a combined hierarchical variable of fentanyl, heroin, and other opioids

^g^ Operationalized as response of “sometimes,” “often,” or “always” to behavior

^h^ Residential treatment is attendance in a residential treatment program, inpatient detox program, or sober house

^i^ Outpatient recovery counseling is attendance of AA/NA meetings or receipt of outpatient counseling for substance use

^j^ Operationalized as response of “never” or “rarely” to behavior of using a tester dose or slow shot

^k^ Operationalized as response of “never” or “rarely” to behavior of using with someone else at the same time

^l^ One missing response

### Bivariate results

Table 2 presents the bivariate results. Compared to White participants, Black participants had higher odds of using alone (OR = 2.17, 95% CI 1.08–4.36, p = 0.03) and lower odds of not using a tester dose (OR = 0.48, 95% CI 0.24–0.93), indicating greater tester dose use. For Latino participants the association estimates for using alone (OR = 1.33, 95% CI 0.60–2.92, p = 0.48) and for a tester dose (OR = 0.64, 95% CI 0.32–1.28, p = 0.21) were in the same direction as Black participants but were not statistically significant. There were no statistically significant differences for using alcohol or benzodiazepines on the same day as opioids across race/ethnicity groups. Of the predisposing and enabling factors considered, being a participant from San Francisco was associated with lower odds of using alcohol or benzodiazepines on the same day as opioids (OR = 0.45, 95% CI: 0.27–0.77, p < 0.01).

**Table 2:** Bivariate association of baseline characteristics of participants with a history of opioid overdose from Boston and San Francisco with overdose risk behaviors of using alcohol or benzodiazepines on the same day as opioids, not using a tester dose, and using alone, 2019—2022 (N = 247)

	Used alcohol or benzodiazepines the same day as opioids OR (95%CI) p-value	Did not use a tester dose OR (95%CI) p-value	Used alone OR (95%CI) p-value
*Independent Variables*			
Race & Ethnicity			
White	Ref	Ref	Ref
Black	0.90 (0.47–1.75) 0.76	0.48 (0.24–0.93) 0.03	2.17 (1.08–4.36) 0.03
Latino	0.81 (0.40–1.64) 0.56	0.64 (0.32–1.28) 0.21	1.33 (0.60–2.92) 0.48
*Predisposing Variables*			
Age for every 10-year increase [median, IQR]	0.94 (0.73–1.21) 0.61	0.80 (0.62–1.03) 0.08	1.20 (0.91–1.60) 0.19
Gender			
Male or trans male	Ref	Ref	Ref
Female or trans female	1.02 (0.61–1.72) 0.93	1.22 (0.73–2.05) 0.45	1.40 (0.79–2.48) 0.25
*Enabling Variables*			
Site			
Boston	Ref	Ref	Ref
San Francisco	0.45 (0.27–0.77) < 0.01	1.38 (0.83–2.29) 0.21	0.78 (0.44–1.38) 0.39
Treated with MOUD ^a,b^	1.45 (0.84–2.51) 0.19	1.49 (0.86–2.57) 0.15	1.23 (0.66–2.29) 0.52
Unstably housed ^a,c^	1.32 (0.78–2.23) 0.31	1.37 (0.81–2.31) 0.24	0.63 (0.36–1.13) 0.12
Incarcerated ^a,d^	0.54 (0.23–1.24) 0.15	1.18 (0.52–2.72) 0.69	0.49 (0.16–1.49) 0.21
Obtained GED or above	1.05 (0.49–2.27) 0.90	0.95 (0.44–2.04) 0.90	0.83 (0.36–1.92) 0.67

^a^ Variable assessed over the past four months

^b^ MOUD defined as taking methadone from a methadone clinic for OUD, taking Suboxone prescribed by a doctor, receiving Sublocade injection by a doctor, receiving Vivitrol injection by a doctor, or taking naltrexone prescribed by a doctor

^c^ Unstable housing operationalized as spending any nights on the street or in a homeless shelter

^d^ Incarceration defined by self-report

### Multivariable results: model 1 (predisposing factors only)

After adjusting for predisposing factors (Table 3), the associations of Black race with using alone (aOR = 2.01, 95% CI 0.99–4.10, p = 0.05) and tester doses (aOR = 0.51, 95% CI 0.26–1.01, p = 0.05) were attenuated, and no longer statistically significant (Table 3). Other analyses remained statistically insignificant.

### Multivariable results: model 2 (predisposing and enabling factors)

In model 2 (Table 3), after adjusting for predisposing and enabling factors there were no statistically significant associations between race or ethnicity and individual overdose risk behaviors. See supplemental Tables 1–3, 4a, and 4b for the full models encompassing all multivariate associations among the independent variables, predisposing and enabling factors, and the outcomes.

**Table 3:** Adjusted multivariable association of race and ethnicity among participants with a history of opioid overdose from Boston and San Francisco with overdose risk behaviors of using alcohol or benzodiazepines on the same day as opioids, not using a tester dose, and using alone, 2019 – 2022 (N = 247)

	Used alcohol or benzodiazepines the same day as opioids aOR (95%CI) p-value	Did not use a tester dose aOR (95%CI) p-value	Used alone aOR (95%CI) p-value
Model 1: Predisposing factors ^a^			
White	Ref	Ref	Ref
Black	0.93 (0.47–1.82) 0.83	0.51 (0.26–1.01) 0.05	2.01 (0.99–4.10) 0.05
Latino	0.81 (0.40–1.64) 0.55	0.63 (0.31–1.29) 0.21	1.37 (0.62–3.04) 0.43
Model 2: Predisposing & enabling factors ^b^			
White	Ref	Ref	Ref
Black	0.89 (0.44–1.78) 0.74	0.56 (0.28–1.13) 0.11	2.05 (0.99–4.26) 0.05
Latino	0.85 (0.40–1.81) 0.68	0.65 (0.31–1.37) 0.26	1.40 (0.61–3.23) 0.43

Model 1 adjusted for age and gender

^b^ Model 2 adjusted for age, gender, site, MOUD treatment, housing, incarceration, and GED receipt

### Secondary analyses

In multivariable analyses (Table 4) that separately examined the outcome of using alcohol or benzodiazepines with opioids separately, Black participants had lower odds of using benzodiazepines on the same day that they used opioids compared to White participants in both the models that adjusted only for predisposing factors (aOR = 0.34, 95% CI 0.16–0.75, p < 0.01) and for both predisposing and enabling factors (aOR = 0.34, 95% CI 0.15–0.76, p < 0.01). All other comparisons were not statistically significant.

**Table 4:** Bivariate and adjusted multivariable association of race and ethnicity among participants with a history of opioid overdose from Boston and San Francisco with overdose risk behaviors of using alcohol on the same day as opioids and using benzodiazepines on the same day as opioids, 2019 – 2022 (N = 247)

	Used alcohol on the same day as opioids aOR (95%CI), p-value	Used benzodiazepines on the same day as opioids aOR (95%CI), p-value
*Bivariate association*		
White	Ref	Ref
Black	1.87 (0.95—3.70) 0.07	0.30 (0.14—0.64) < 0.01
Latino	0.80 (0.35—1.81) 0.59	0.75 (0.37—1.52) 0.42
*Model 1: Predisposing factors*		
White	Ref	Ref
Black	1.60 (0.79—3.24) 0.19	0.34 (0.16—0.75) < 0.01
Latino	0.80 (0.35—1.84) 0.60	0.72 (0.35—1.48) 0.37
*Model 2: Predisposing and enabling factors*		
White	Ref	Ref
Black	1.63 (0.80—3.35) 0.18	0.34 (0.15—0.76) < 0.01
Latino	0.72 (0.30—1.74) 0.47	0.72 (0.33—1.56) 0.41

^a^ Model 1 adjusted for age and gender

^b^ Model 2 adjusted for age, gender, site, MOUD treatment, housing, incarceration, and GED receipt

## Discussion

In this study of participants with opioid use disorder and a history of overdose, using alcohol and benzodiazepines on the same day as opioids, not using a tester dose, and using alone were common overdose risk behaviors. Statistically significant differences found in unadjusted analyses comparing Black and White overdose survivors, where Black participants had higher odds of using alone and tester dose use, were no longer significant after adjusting for age, gender, and structural determinants of health, including housing, incarceration, education, and MOUD treatment. Black participants had lower adjusted odds of using benzodiazepines when using opioids compared to White participants in the secondary analysis.

The association observed between Black compared to White participants and using alone (OR = 2.17, 95% CI 1.08–4.36) and not using a tester dose (OR = 0.48, 95% CI 0.24–0.93) in the unadjusted analyses reflected different frequencies of overdose prevention behaviors. Our study was a secondary analysis of data from an interventional cohort study of people who all had a history of overdose. The fact that all participants had overdose experiences suggests that both the burden of overdose risk and the use of protective behaviors were substantively enhanced across racial and ethnic groups [6, 31]. After adjusting for housing, incarceration, education, and MOUD treatment, which are manifestations of structural racism, the findings were no longer significant. The varying unadjusted frequencies of overdose risk behaviors by race may reflect different strategies for coping with the structural determinants of health faced by overdose survivors who continue to use opioids. For example, social and economic marginalization may limit Black people who use drugs from accessing communal drug-use spaces, while fears of disproportionate surveillance and policing may further reinforce their decision to use drugs alone [32]. Thus, Black overdose survivors who use drugs alone may adapt by testing their drug doses more frequently to reduce the risk of overdose. Our findings suggest efforts to address overdose risk for people who use drugs require more than risk reduction education, and should incorporate social justice approaches that address structural determinants of health, such as housing, incarceration, and access to MOUD [33].

We did not detect statistically significant differences in risk behaviors by Latino ethnicity, which may be partly attributable to limited statistical power. Future studies should continue to explore opioid overdose risk behaviors among Latino communities in greater detail, including through recruitment and selection of protocols that facilitate Latino engagement in research. Such strategies might include greater engagement of community organizations that serve Latino populations, as well as bicultural and bilingual research staff who can facilitate rapport-building with monolingual Spanish speakers and/or those who prefer to communicate in Spanish [34–37].

We found Black participants to have lower odds of using benzodiazepines on the same day that they used opioids. However, that finding may have been counterbalanced by possibly higher odds of using alcohol on the same day as opioids; although this finding was not significant, prior investigators have found higher rates of deaths attributed to alcohol and opioids together among Black people. [7] Given the similar pharmacology of benzodiazepines and alcohol, and the similar overdose risk of using these drugs with opioids, we combined those two classes for analysis, which may have canceled out differences in substance use. Black patients are less likely to receive prescriptions for benzodiazepines, [38, 39] an example of racial bias in medical care that might be paradoxically protective by reducing co-use with opioids. Though providers often counsel about the risks of alcohol and benzodiazepine use with opioids, our analyses suggest that substance-specific recommendations may be needed, and future research with Black people who report using alcohol and opioids should further inform these recommendations.

This study must be interpreted in the context of several limitations. First, our assessment of engagement in risk behaviors was based on self-report data and is therefore subject to recall and social desirability biases. We conducted a cross-sectional analysis, so risk behaviors could have preceded outcomes that were also measured over the last four months. Next, the exclusion of monolingual Spanish-speaking participants may have biased our results comparing behaviors among Latino to White participants toward the null, as data has shown that numerous health outcomes are worse among Spanish-speaking compared to English-speaking Latino people [40]. Similarly, overdose risk varies substantially by specific Latino ethnic identities [41]. These differences may be obscured when Latino ethnicities are aggregated as we did in this study. In general, the REBOOT study was not designed to detect differences by race or ethnicity, thus the recruitment strategy and sample size may have contributed to the null findings. In future studies examining racial and/or ethnic variation in substance use risk behaviors, adequate statistical power and tailored recruitment strategies are warranted. Furthermore, this study drew participants from the REBOOT 2.0 cohort, which was based in Boston and San Francisco, two urban cities that are relatively well-resourced in terms of harm reduction services, and eligibility for the study required prior naloxone receipt. Because naloxone receipt may indicate prior engagement with harm reduction services, this requirement may have selected for participants who were already more likely to engage in protective behaviors. Thus, our findings may underestimate the frequency of deleterious overdose risk behaviors among other populations with opioid use disorder, such as those living in rural areas [42], those who have not used naloxone, or those who are not willing to participate in research [43]. Finally, this analysis focused on identifying racial and ethnic differences using three mutually exclusive racial/ethnic groups, excluding participants who did not self-identify with these groups. We did not explore differences in risk behaviors among American Indian and Alaskan Native people due to the low enrollment of individuals from these groups in the parent study. Future overdose risk studies should be designed to focus on American Indian and Alaskan Native populations given their high rates of overdose deaths [44].

## Conclusions

After adjusting for structural determinants in a cohort of people who had survived an opioid overdose, we did not identify significant differences in opioid overdose risk behaviors among Black or Latino participants compared to White participants. These findings suggest that structural barriers may drive observed racial and ethnic disparities in overdose risk more than individual behavior differences. Addressing structural barriers related to housing and access to services that disproportionately affect Black and Latino people, alongside harm reduction education and services tailored to these communities, may be important for mitigating known racial and ethnic disparities in overdose risk.

## Supplementary Information

Below is the link to the electronic supplementary material.

Supplementary Material 1

## Data Availability

The data that support the findings of this study are not publicly available but available upon request from the authors, subject to restrictions from the institutional review boards' protections of research participants' privacy.

## Abbreviations

AA/NA: Alcoholics Anonymous / Narcotics Anonymous
BMHSU: Behavioral Model of Health Services Use
GED: General Education Diploma
HIV: Human Immunodeficiency Virus
MOUD: Medications for opioid use disorder
OUD: Opioid use disorder
REBOOT 2.0 Study: REpeated dose Behavioral intervention to reduce Opioid Overdose

## Footnotes

1 In this paper, we use the term Black to refer to non-Latino Black and/or African American individuals and the term White to refer to non-Latino White individuals. We use the term Latino to refer to Hispanic and/or Latino/a/e/x individuals (1).
