# Supplementary material for: A comparison of opioid overdose risk behaviors by race and ethnicity among overdose survivors in Boston, MA, and San Francisco, CA
Source: Harm Reduct J. 2026 Apr 20;23:97. doi: 10.1186/s12954-026-01451-9 (PMC13227638; doi:10.1186/s12954-026-01451-9)
Supplement: Supplementary file 1 — Supplementary Material 1 [file 12954_2026_1451_MOESM1_ESM.docx]

**Supplementary Tables**

**Supplementary Table 1.** Adjusted multivariable association of baseline characteristics of participants with a history of opioid overdose from Boston and San Francisco with overdose risk behavior of using alcohol and/or benzodiazepines on the same day as opioids, 2019 – 2022 (N = 246).

| Characteristics | Model 1: Predisposing factors only  aOR (95%CI) | Model 2: Predisposing and enabling factors  aOR (95% CI) |
| --- | --- | --- |
| Race & Ethnicity  White  Black  Latine | Ref  0.93 (0.47 - 1.82)  0.81 (0.40 - 1.64) | Ref  0.89 (0.44 - 1.78)  0.85 (0.40 - 1.81) |
| Age for every 10-year increase [median, IQR] | 0.94 (0.73 - 1.21) | 0.91 (0.69 - 1.19) |
| Gender  Male or trans male  Female or trans female | Ref  1.01 (0.60 - 1.71) | Ref  0.93 (0.54 - 1.62) |
| Site  Boston  San Francisco |  | Ref  0.47 (0.26 - 0.85) |
| Treated with MOUD ^a,b^ |  | 1.24 (0.68 - 2.24) |
| Unstably housed ^a,c^ |  | 1.06 (0.59 - 1.90) |
| Incarcerated ^a,d^ |  | 0.52 (0.22 - 1.22) |
| Obtained GED or above |  | 1.04 (0.45 - 2.40) |

^a^ Variable assessed over the past four months.

^b^ MOUD defined as taking methadone from a methadone clinic for OUD, taking Suboxone prescribed by a doctor, receiving Sublocade injection by a doctor, receiving Vivitrol injection by a doctor, or taking naltrexone prescribed by a doctor.

^c^ Unstable housing operationalized as spending any nights on the street or in a homeless shelter.

^d^ Incarceration defined by self-report.

**Supplementary Table 2.** Adjusted multivariable association of baseline characteristics of participants with a history of opioid overdose from Boston and San Francisco with overdose risk behavior of not using a tester dose, 2019 – 2022 (N = 247).

| Characteristics | Model 1: Predisposing factors only  aOR (95%CI) | Model 2: Predisposing and enabling factors  aOR (95%CI) |
| --- | --- | --- |
| Race & Ethnicity  White  Black  Latine | Ref  0.51 (0.26 - 1.01)  0.63 (0.31 - 1.29) | Ref  0.56 (0.28 - 1.13)  0.65 (0.31 - 1.37) |
| Age for every 10-year increase [median, IQR] | 0.82 (0.64 - 1.07) | 0.81 (0.62 - 1.07) |
| Gender  Male or trans male  Female or trans female | Ref  1.23 (0.73 - 2.08) | Ref  1.45 (0.83 - 2.50) |
| Site  Boston  San Francisco |  | Ref  1.99 (1.10 – 3.62) |
| Treated with MOUD ^a,b^ |  | 1.92 (1.05 - 3.52) |
| Unstably housed ^a,c^ |  | 1.79 (0.99 - 3.25) |
| Incarcerated ^a,d^ |  | 1.04 (0.43 - 2.49) |
| Obtained GED or above |  | 0.81 (0.35 - 1.86) |

^a^ Variable assessed over the past four months.

^b^ MOUD defined as taking methadone from a methadone clinic for OUD, taking Suboxone prescribed by a doctor, receiving Sublocade injection by a doctor, receiving Vivitrol injection by a doctor, or taking naltrexone prescribed by a doctor.

^c^ Unstable housing operationalized as spending any nights on the street or in a homeless shelter.

^d^ Incarceration defined by self-report.

**Supplementary Table 3.** Adjusted multivariable association of baseline characteristics of participants with a history of opioid overdose from Boston and San Francisco with overdose risk behavior of using alone, 2019 – 2022 (N = 247).

| Characteristics | Model 1: Predisposing factors only  aOR (95%CI) | Model 2: Predisposing and enabling factors  aOR (95%CI) |
| --- | --- | --- |
| Race & Ethnicity  White  Black  Latine | Ref  2.01 (0.99 - 4.10)  1.37 (0.62 - 3.04) | Ref  2.05 (0.99 - 4.26)  1.40 (0.61 - 3.23) |
| Age for every 10-year increase [median, IQR] | 1.16 (0.87 - 1.54) | 1.09 (0.81 - 1.46) |
| Gender  Male or trans male  Female or trans female | Ref  1.39 (0.78 - 2.49) | Ref  1.28 (0.70 - 2.33) |
| Site  Boston  San Francisco |  | Ref  0.74 (0.39 - 1.43) |
| Treated with MOUD ^a,b^ |  | 1.15 (0.58 - 2.28) |
| Unstably housed ^a,c^ |  | 0.62 (0.33 - 1.19) |
| Incarcerated ^a,d^ |  | 0.52 (0.17 - 1.63) |
| Obtained GED or above |  | 0.91 (0.37 - 2.24) |

^a^ Variable assessed over the past four months.

^b^ MOUD defined as taking methadone from a methadone clinic for OUD, taking Suboxone prescribed by a doctor, receiving Sublocade injection by a doctor, receiving Vivitrol injection by a doctor, or taking naltrexone prescribed by a doctor.

^c^ Unstable housing operationalized as spending any nights on the street or in a homeless shelter.

^d^ Incarceration defined by self-report.

**Supplementary Table 4a.** Bivariate association of baseline characteristics of participants with a history of opioid overdose from Boston and San Francisco with overdose risk behavior of using alcohol on the same day as opioids, 2019 – 2022 (N = 246).

| Characteristics | Model 1: Predisposing factors only  OR (95%CI) | Model 2: Predisposing and enabling factors  OR (95%CI) |
| --- | --- | --- |
| Race & Ethnicity  White  Black  Latine | Ref  1.60 (0.79 - 3.24)  0.80 (0.35 - 1.84) | Ref  1.63 (0.80 - 3.35)  0.72 (0.30 - 1.74) |
| Age for every 10-year increase [median, IQR] | 1.51 (1.14 - 2.02) | 1.53 (1.13 - 2.06) |
| Gender  Male or trans male  Female or trans female | Ref  0.81 (0.45 - 1.46) | Ref  0.81 (0.44 - 1.48) 0.49 |
| Site  Boston  San Francisco |  | Ref  0.94 (0.50 - 1.77) |
| Treated with MOUD ^a,b^ |  | 0.93 (0.48 - 1.82) |
| Unstably housed ^a,c^ |  | 1.50 (0.77 - 2.90) |
| Incarcerated ^a,d^ |  | 0.64 (0.22 - 1.88) |

^a^ Variable assessed over the past four months.

^b^ MOUD defined as taking methadone from a methadone clinic for OUD, taking Suboxone prescribed by a doctor, receiving Sublocade injection by a doctor, receiving Vivitrol injection by a doctor, or taking naltrexone prescribed by a doctor.

^c^ Unstable housing operationalized as spending any nights on the street or in a homeless shelter.

^d^ Incarceration defined by self-report.

**Supplementary Table 4b.** Adjusted multivariable association of baseline characteristics of participants with a history of opioid overdose from Boston and San Francisco with overdose risk behavior of using benzodiazepines on the same day as opioids, 2019 – 2022 (N = 247).

| Characteristics | Model 1: Predisposing factors only  aOR (95%CI) | Model 2: Predisposing and enabling factors  aOR (95%CI) |
| --- | --- | --- |
| Race & Ethnicity  White  Black  Latine | Ref  0.34 (0.16 - 0.75)  0.72 (0.35 - 1.48) | Ref  0.34 (0.15 - 0.76)  0.72 (0.33 - 1.56) |
| Age for every 10-year increase [median, IQR] | 0.65 (0.49 - 0.86) | 0.61 (0.45 - 0.81) |
| Gender  Male or trans male  Female or trans female | Ref  0.97 (0.56 - 1.67) | Ref  0.90 (0.51 - 1.59) |
| Site  Boston  San Francisco |  | Ref  0.57 (0.31 - 1.05) |
| Treated with MOUD ^a,b^ |  | 1.71 (0.91 - 3.22) |
| Unstably housed ^a,c^ |  | 1.01 (0.55 - 1.87) |

^a^ Variable assessed over the past four months.

^b^ MOUD defined as taking methadone from a methadone clinic for OUD, taking Suboxone prescribed by a doctor, receiving Sublocade injection by a doctor, receiving Vivitrol injection by a doctor, or taking naltrexone prescribed by a doctor.

^c^ Unstable housing operationalized as spending any nights on the street or in a homeless shelter.
